# From Inventories to Insights: Environmental Gradients Structuring Macro‐Moths Assemblages Recorded in Nature Reserves

**DOI:** 10.1002/ece3.73788

**Published:** 2026-06-08

**Authors:** Zuzana Kubincová, Zdeněk Faltýnek Fric, Radek Hejda, Martin Konvička, Lukáš Spitzer, Alena Sucháčková, Pavel Vrba, Jan Walter

**Affiliations:** ^1^ Department of Biology, Geosciences and Environmental Education, Faculty of Education University of West Bohemia Pilsen Czech Republic; ^2^ Institute of Entomology Biological Centre CAS Ceske Budejovice Czech Republic; ^3^ Faculty of Agrobiology Food and Natural Resources Praha‐Suchdol Czech Republic; ^4^ Nature Conservation Agency of the Czech Republic Praha 11‐Chodov Czech Republic; ^5^ Faculty of Science University of South Bohemia Ceske Budejovice Czech Republic; ^6^ Muzeum Regionu Valašsko Vsetín Czech Republic; ^7^ Senckenberg German Entomological Institute Müncheberg Germany; ^8^ Zoological Department Museum of West Bohemia Pilsen Czech Republic

**Keywords:** biodiversity conservation, landscape ecology, lepidoptera, life histories, protected areas, specialists, species traits

## Abstract

Inventorying biodiversity in reserves is a routine activity in conservation, yet for hyper‐diverse insect groups such as macro‐moths, general ecological patterns remain underexplored. We collated and analysed macro‐moths (Lepidoptera) inventories from 292 nature reserves across the Czech Republic, encompassing 941 species. Inventories varied in surveying effort and methodology but collectively allowed examination of factors influencing moths' species richness, assemblages' composition and their associations with the species' traits. Species richness increased with elevational scope and was highest in reserves covering steppes and mesic grasslands. Geographic position of the reserves, altitude and elevational scope affected the moth assemblages' composition. Reserves at low altitudes hosted larger moths forming multiple generations and developing on forbs, whereas high‐altitude reserves hosted species developing on shrubs and trees, forming fewer generations, overwintering in later stages and inhabiting broader habitat and altitude ranges. Regional biogeography also affected the assemblages, with Carpatho‐Pannonian reserves supporting large‐winged habitat‐specialists, often overwintering in early developmental stages. Our results demonstrate that even heterogeneous data can reveal consistent patterns when interpreted through species' traits. These findings demonstrate the value of reserve inventories compiled by dedicated amateur lepidopterists for deciding on reserve management strategies and identifying key habitats for species of conservation concern.

## Graphical abstract

The article analyses macro‐moth (Lepidoptera) inventory data from 292 nature reserves across the Czech Republic, comprising 941 species. It examines how reserve characteristics, geographic position and altitude influence species richness, community composition and their associations with species traits. The results show that even heterogeneous inventory data can reveal consistent macroecological patterns and provide valuable insights for biodiversity conservation and reserve management.

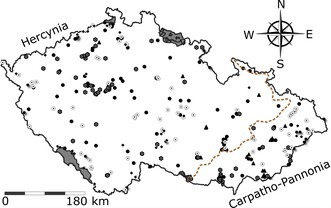

## 1 Introduction

Inventorying the biota of protected areas is an incessant activity of conservationists, both in Europe and worldwide (Cogălniceanu et al. 2025; Shuey 2005; Ulrich et al. 2025). Inventories inform relevant stakeholders on biodiversity values of specific sites, allow comparisons in time and guide management measures (Mota et al. 2023). For certain taxonomic groups, mere species lists may directly inform on the state of the habitats and necessity of eventual interventions. This applies to plants and vegetation, as many reserves have been established with the aim to preserve specific vegetation types (Holz et al. 2022; Horák et al. 2019). In animals, this applies to such well‐studied and species‐poor groups as large mammals (Hayward 2011; Lahkar et al. 2020), birds (Hochkirch et al. 2023; Leitão et al. 2022), cold‐blooded tetrapods (Azam et al. 2016; Ficetola et al. 2015), but also to model insect groups such as butterflies (Bartonova et al. 2016; Habel et al. 2024; McLean et al. 1995). In contrast, some arthropod groups are so species rich that inventory results evade straightforward interpretations (Potapov et al. 2020).

Such a hyperdiverse group is represented by the (night‐active) macrolepidoptera, or macro‐moths, a traditional paraphyletic grouping (Potocký et al. 2018), which is ≈7× more species‐rich than the monophyletic butterflies in Central Europe (Laštůvka et al. 2023). Macro‐moths occupy all terrestrial habitats and exhibit a wider range of life histories than butterflies (cf. Dar and Jamal 2021; Thorn et al. 2015). Although comprehensive identification literature exists for many regions (e.g., Macek et al. 2007, 2008, 2012) and there is a venerable tradition of inventorying and classifying moths' communities (e.g., Bergmann 1954; Piccini et al. 2023; Spitzer et al. 1999), generalisations of the moths' ecology, distribution and abundance patterns lag behind many other insect groups due to their nocturnal habits, taxonomic complexity and difficulty of identifying certain species (Mironov 2003; Sisson et al. 2023). Still, studies from many regions have reported significant declines in moth abundance and diversity (Burner et al. 2021; Conrad et al. 2006; Percel et al. 2025; Wagner et al. 2021), attributable to similar factors that are threatening insects worldwide (Fox 2012). For deeper understanding of moths' fauna changes, however, large and ideally long‐term data are necessary. Reserve inventories, in contrast, provide just snapshot pictures of assemblages' composition in the form of species lists, sometimes supplemented by relative abundances.

A tool for searching general patterns in species lists is analysis of species traits (herein ‘traits’), connecting species identities, life strategies and environments (Bartonova et al. 2016; McGill et al. 2006; Végvári et al. 2014; Wong et al. 2019). Regarding moths, trait analyses have been used, e.g., for identifying the predictors of extinctions' propensity (Mattila et al. 2006, 2008, 2009), colonisation ability (Summerville 2015), or species' responses to landscape fragmentation (Ewers and Didham 2006; Franzén et al. 2012; Prugh et al. 2008). Moreover, moths' traits can be grouped into ecologically interpretable units reflecting vegetation type, host plant associations or landscape characteristics (Szanyi et al. 2016).

In Europe, protected areas are routinely divided into large‐scale and small‐scale. While the former aim to conserve entire functional landscape units (mountain ranges, river deltas, canyons, etc.), the latter are established to protect specific biotopes or rare species (Laguna et al. 2004; Slancarova et al. 2014; Volenec and Dobson 2020). In the Czech Republic, a medium‐sized European country, in addition to 31 large‐scale areas (4 National Parks, 27 Protected Landscape Areas), there are 2680 smaller‐scale protected units, ranging in area from < 1 ha to 29 km^2^. These have been established by diverse authorities under several legal categories, sometimes within the large‐size units (Pyšek et al. 2002). We hereinafter label them all as ‘reserves’.

To assess which ecological and conservation‐relevant insights can be derived from macro‐moth inventories of these reserves, we collated presence–absence data produced during reserve inventories across the Czech Republic and analysed them in relation to reserve characteristics and species traits. We tested two main hypotheses: (H1) The macro‐moth assemblages recorded in reserves are structured by characteristics related to geography positions, spatial extent, environmental heterogeneity and major biotopes of the reserves. Resulting gradients in macro‐moth species composition are associated with consistent shifts in species traits and the representation of Red‐listed species. (H2) Interpreting responses in macro‐moth traits by environmental conditions of the reserves can reveal robust macroecological patterns relevant for biodiversity conservation and reserve management.

## 2 Material and Methods

### 2.1 Data Origin

**FIGURE 1:**
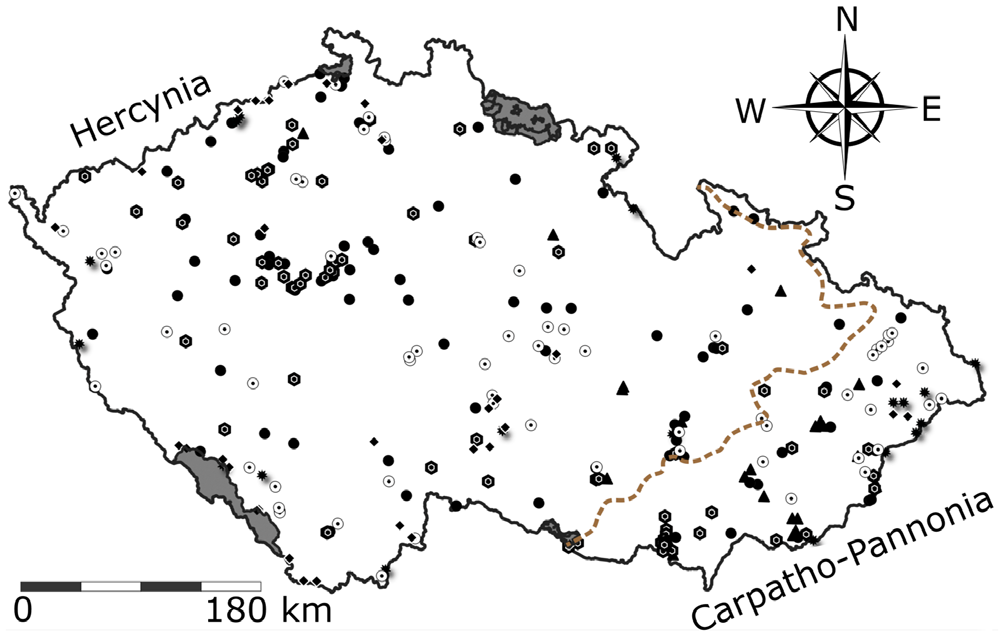
Locations of the 292 Czech Republic reserves included in the study of moths' traits. Dark shading shows the four National Parks and the dashed line divides the Hercynian and Carpatho‐Pannonian regions. Symbols indicate the biotope prevailing in the reserves: Black stars—mountain forest, black circle—forest, white circle with black dot—wetland, black rhombus—peat bog, black triangle—mesic grassland, hexagon with a black dot—steppe.

The Czech Republic is a landlocked country of 78,870 km^2^, situated within the nemoral vegetation zone. The climate is temperate, with four distinct seasons, warm summers and cold snowy winters (Brázdil et al. 2022). Its western part, shaped by the Variscan orogeny, consists of gently rolling hills only locally reaching the timberline (Krkonoše and Jeseník Mts., 1603 and 1491 m a.s.l.) and is dissected by narrow stream canyons or wider river plains. The eastern part of the country belongs to the Carpatho‐Pannonian region with steeper mountains (max. 1323 m a.s.l.) and a more direct connection to warmer southeastern Europe (Figure 1).

The data originated from inventories of macro‐moths in 292 reserves (Supporting Information), commissioned at various occasions by the Nature Conservation Agency of the Czech Republic or Regional Authorities, and carried out by dozens of mainly amateur lepidopterologists since 1990 onwards. The inventoried reserves are scattered across the country, except for the National Parks (Figure 1), and encompass a wide range of mean altitudes (mean 460 m, from 116 to 1155 m), reserve areas (mean 183 ha, range from 0.33 to 2029 ha). They include practically all habitat types present in the country. We retrieved the inventories' data either from the Information System of Nature Conservation managed by the Nature Conservation Agency of the Czech Republic or from unpublished manuscripts deposited at Regional Authorities. We used only inventories that explicitly targeted macro‐moths and employed appropriate sampling methods.

The inventories were typically short‐term, conducted within a single season or over a few consecutive years. They produced species lists of macro‐moths recorded in each reserve. Besides this, they were executed very heterogeneously regarding the light source used, numbers of trapping nights and traps set per night, spatial placement of the traps and seasonal coverage. Regretfully, these details were not consistently reported and therefore cannot be explicitly tested and modelled. We therefore use below only a simple proxy of surveying effort. Furthermore, we included only comprehensive inventories that were concluded with a final report or a regionally published scientific paper, rather than the results of an opportunistic visit to a reserve and excluded studies with too low numbers (< 35) of reported species.

### 2.2 Reserve Characteristics

Each reserve was characterised by area, geography coordinates (latitude, longitude and altitude) and elevational scope (difference between maximum and minimum altitude within the reserve as a proxy for topographic heterogeneity). We also coded the position of each reserve in the phytogeographic regions of the Czech Republic (Skalický 1988), a scheme distinguishing Hercynian and Carpatho‐Pannonian divisions, and thermophytic, mesophytic and oreophytic vegetation regions. Finally, we characterised each reserve by the main biotope type, broadly categorised into steppe grasslands and shrubs (including xeric slopes of river canyons), mesic grasslands, eutrophic wetlands, peat bogs (including subalpine grasslands), lowland to midland forests and mountain forests (cf. Chytrý et al. 2010). As a reserve may contain several biotope types, we classified them according to the one locally deemed most valuable by reserve managers. The moths' nomenclature follows Laštůvka et al. (2023).

### 2.3 Species Traits

We used 11 traits arguably connected to moth dispersal, larval and adult feeding, development length, survival abilities and niche breadth. Most species traits were adopted from Potocký et al. (2018), which explicitly targeted the situation in Central Europe and in cases of polymorphic traits' states, coded the situation for lowlands of the Czech Republic. For a few species not covered by Potocký et al. (2018), we extracted the traits from literature covering identical region (Macek et al. 2007, 2008, 2012). All traits were treated as species‐level characteristics and expressed using standardised values: (1) wingspan (forewing length in cm); (2) overwintering stage (ranked 1 to 4; eggs to adults); (3) voltinism (number of generations per year); (4) migration ability (1/0; yes/no); (5) flight period length (sum (1–5) of the following flight periods: early spring, spring, summer, autumn, winter); (6) trophic range (1– monophages; 2—oligophages, 3—polyphages); (7) host plant form (ranked: 1—forbs; 2—grasses; 3—shrubs; 4—trees; 5—non‐vascular plants such as lichens, mosses, fungi etc.); (8) host plant part (factor with 3 levels: flower/seeds, leaves, stems/roots); (9) larval sociality (ranked: 1—solitary; 2—small groups in at least some instars; 3—large aggregations in at least some instars); (10) habitat range (1–12, the number of habitat types listed for moths' species according to Macek et al. (2007): xerophilous1, xerophilous2, xerophilous3, hygrophilous1, hygrophilous 2, mesophilous1, mesophilous2, mesophilous3, hygrothermophilous, tyrphophilous, alpine, ubiquitous) and (11) altitude range (1–3; the number of altitudinal bands (< 250, 250–1000, > 1000 m a.s.l.) inhabited by the species). We also extracted threat status according to the national Red list (Hejda et al. 2017) and coded them by increasing threat level (0—LC (least concern), 1—NT (near threatened), 2—VU (vulnerable), 3—EN (endangered), 4—CR (critically endangered), 5—RE (regionally extinct)).

### 2.4 Statistical Analysis

The large and often unreported variation in light‐trapping methods, numbers of light traps set per night, or combination of various techniques required a control for surveying effort. We used a simple ordinal proxy, surveying effort, characterising each inventory by the total number of visits per reserve, using a simple scale: 1 (≤ 5 visits), 2 (6–10 visits) and 3 (> 10 visits). A visit was defined as a single‐night light‐trapping event, irrespective of methods applied (light sheet or portable light traps), typically lasting several evening hours. This proxy was highly significantly related to number of recorded species (linear regression, number of species ~109.8 (±12.83 SE) + 45.1 (±7.63 SE) surveying efforts, *F*_(1,290df)_ = 35.05, *p* < 0.001) but left sufficient unexplained variation (*R* ^2^ = 0.105) for further modelling. We also subjected the surveying effort values to logistic ordinal regression, using reserve characteristics as predictors (Christensen 2025), finding that only two reserve characteristics, area (negatively) and elevational scope (positively) were correlated with surveying effort (Table S1).

To analyse patterns influencing the number of macro‐moth species reported per reserves, we fitted a multiple linear regression model with surveying effort (characterised by the ordinal 1–3 scale), all the reserve characteristics (i.e., reserve area, altitude, elevational scope, longitude and latitude, phytogeographic region and division, and biotope type). This model allowed simultaneous assessment of separate effects of individual predictors within a single analytical framework. Next, we subsequently removed all the nominally non‐significant predictors to attain a simplified model with all predictors different from each other and nominally significant at *p* < 0.05 level. The analysis was carried out in R (R Core Team 2025), with both predictors and the response variable standardised to zero mean and unit variance prior to the modelling.

To investigate the separate effects of reserve characteristics on moth species' composition, and to interpret these effects by the moths' traits, we followed the fourth‐corner approach (Dray et al. 2014; Legendre et al. 1997), which relates constrained species scores obtained by ordination of two tables, in our case the species~ reserves characteristics, to a third table, in our case the traits. We used CANOCO 5 (Šmilauer and Lepš 2014) for the calculations. In this two‐step process, the first step was the unimodal detrended canonical correspondence analysis (DCCA); we selected the detrending option to eliminate arch effects (ter Braak 1986), apparent if detrending was not applied. The sequentially tested reserves' characteristics were area, altitude, elevational scope, geography (based on a forward selection from latitude, longitude and altitude of the reserves, their polynomials and multiplicative interactions), phytogeographic regions and divisions, and biotopes. DCCA significances were evaluated using 999 Monte Carlo permutations. In the second step, constrained species scores of the statistically significant (*p* ≤ 0.05) DCCAs were subjected to redundancy analysis, RDA, a linear multivariate method, relating them to the species' traits. We used forward selection to select the best‐fitting combinations of traits and again evaluated the significances using 999 Monte Carlo permutations.

For visualisation, we used ordination triplots to show the relationships between traits and the significant focal predictors within the ordination space. We determined the gradient of Axis 1 (and Axis 2 where applicable) from the first step. The best‐fitting moth species were projected into the ordination diagrams, and only significant traits were depicted.

## 3 Results

The 292 inventories returned presence data on 941 species, or 86.6% of the 1087 macro‐moths species recorded in the Czech Republic (cf. Laštůvka et al. 2023) (Data S1), the average inventory contained 177.3 ± 106.00 SD species (median 158.5, range 36–589). Fifty inventories (17%) were based on > 10 recording visits, 45 (15%) on 5–10 visits and the majority (197, or 67%) on ≤ 5 visits.

The most frequent species recorded were *Noctua pronuba* (*n* = 252 reserves), *Xestia c‐nigrum* (*n* = 250), *Autographa gamma* (*n* = 245), *Hypena proboscidalis* (*n* = 232) and *Apamea monoglypha* (*n* = 220). In contrast, 46 species were reported from a single reserve. The highest species numbers were reported from large reserves protecting steppe patches amidst thermophilous deciduous forests: Karlštejn (1556 ha, 598 species), Koda (498 ha, 528 species), Mohelenská hadcová step (109 ha, 483 species), Údolí Brtnice (67 ha, 469 species) and from the woodland reserve Strádovské peklo (88 ha, 483 species).

Total of 170 Red‐listed species, that is, 74.2% of the total number of 229 macro‐moths species Red‐listed in the Czech Republic (cf. Hejda et al. 2017), were recorded. The highest numbers were from the following five steppe reserves: Karlštejn (1556 ha, 53 species), Tabulová (108 ha, 49 species), Koda (498 ha, 48 species), Děvín (391 ha, 46 species) and Údolí Říčky (115 ha, 38 species). These reserves also represented the most frequently visited localities, with the highest numbers of visits.

**FIGURE 2:**
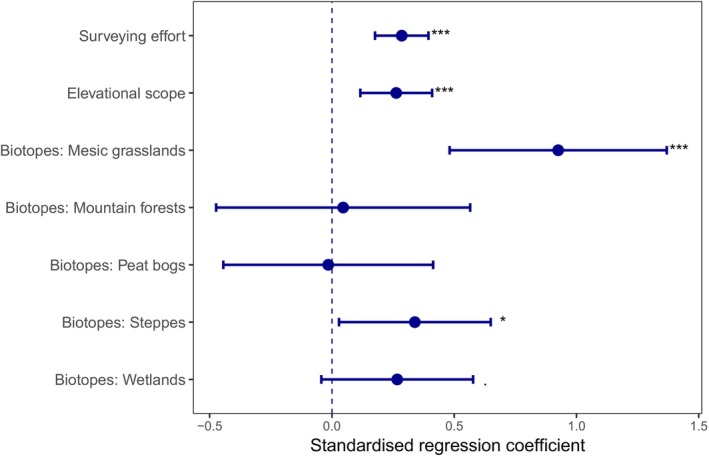
Standardised effects of reserve characteristics on macro‐moth species richness derived from a multiple linear regression model after removing nominally nonsignificant predictors. Points represent standardised regression coefficients, and horizontal bars indicate 95% confidence intervals. The dashed vertical line denotes zero effect. Asterisks indicate statistically significant effects (*p < 0.05, ***p < 0.001). All continuous predictors were standardised to zero mean and unit variance prior to analysis.

The multiple linear regression model explained 22.3% of the variation in species richness (*F*_14,277_ = 5.66, *p* < 0.001, Figure S1). The analysis revealed that the strongest positive predictor of moth species richness was surveying effort. Further significant predictors were elevational scope and biotope, while altitude exhibited a marginally negative effect. Mesic grasslands and steppes supported significantly more species than other biotopes. Area, geography and phytogeographic regions and divisions had no significant effects, but indicated trends towards higher species numbers in larger, easterly situated reserves. Removal of nominally non‐significant predictors resulted in model explaining 20.5% of the variation (*F*_7,284_ = 10.43, *p* < 0.001) (Table 1, Figure 2).

**TABLE 1:** Results of a multiple linear regression model simultaneously assessing effects of all reserve characteristics on recorded macro‐moths species richness across 292 nature reserves (left), and significant model terms retained after removing nominally nonsignificant predictors.

Focal predictor	Simultaneous test of all predictors				Test with significant predictors			
	Estimate	SE	*t*‐value	*p*	Estimate	SE	*t*‐value	*p*
Surveying effort	0.29	0.056	5.16	***	0.29	0.055	5.39	***
Area	0.06	0.064	0.92	NS	—	—	—	—
Altitude	−0.17	0.091	−1.88	(*)	—	—	—	—
Elevational scope	0.22	0.074	2.94	**	0.21	0.058	3.67	***
Longitude	−0.04	0.089	−0.43	NS	—	—	—	—
Latitude	−0.09	0.067	−1.35	NS	—	—	—	—
Phytogeographic region: Oreophyticum	0.11	0.213	0.5	NS	—	—	—	—
Phytogeographic region: Thermophyticum	−0.08	0.156	−0.51	NS	—	—	—	—
Phytogeographic division: Hercynia	−0.21	0.198	−1.07	NS	—	—	—	—
Biotope: mesic grasslands	0.90	0.227	3.95	***	0.82	0.218	3.77	***
Biotope: steppes	0.35	0.159	2.18	*	0.35	0.147	2.36	*
Biotope: mountain forests	0.01	0.266	0.03	NS	−0.21	0.234	−0.93	NS
Biotope: peat bogs	−0.06	0.219	−0.27	NS	−0.11	0.188	−0.60	NS
Biotope: wetlands	0.24	0.159	1.52	NS	0.25	0.153	1.63	NS

*Note:* Species richness and all continuous focal predictors were standardised to zero mean and unit variance prior to analysis. Surveying effort was included as an ordinal proxy based on the number of visits per reserve. Biotope and phytogeographic regions and divisions are shown relative to the reference levels. Asterisks indicate statistically significant effects (*p < 0.05, **p < 0.01, ***p < 0.001, (*) marginally significant).

**FIGURE 3:**
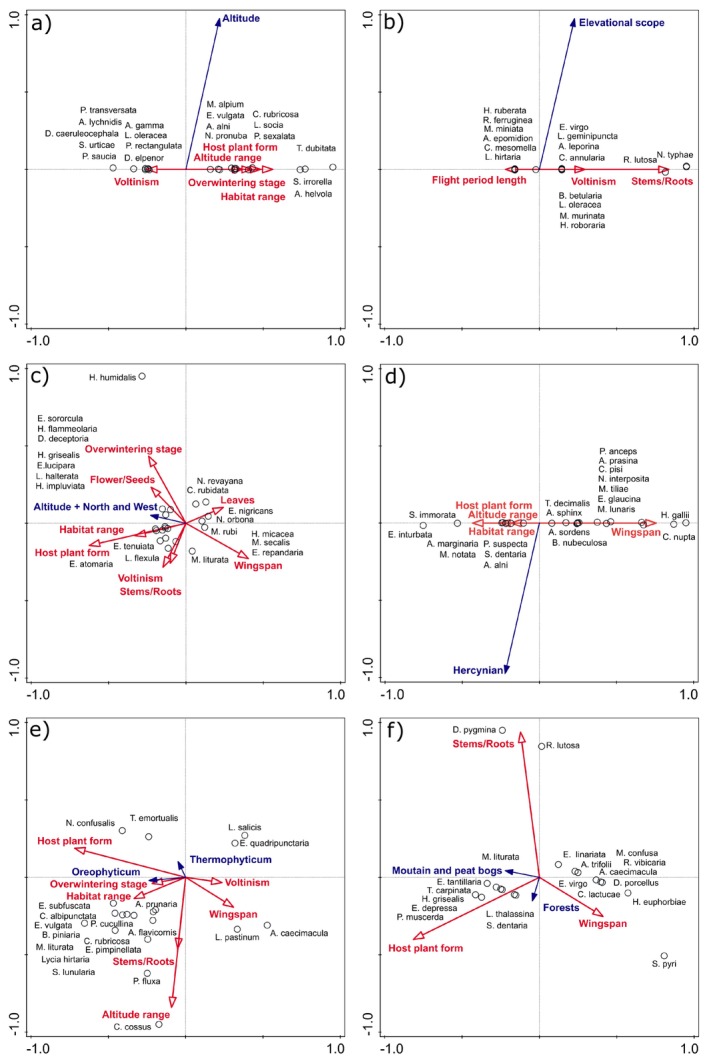
Triplots obtained by redundancy analyses (RDA) relating DCCA species scores of moths constrained by reserve characteristic to species traits of the moths (i.e., Step 2 from the analyses summarised in Table 2). Only traits selected as significant by forward selection are depicted. Focal predictors are in ordination space visualisation are (a) altitude, (b) elevational scope, (c) geography, (d) phytogeographic regions, (e) phytogeographic divisions and (f) biotope. The blue arrows indicate the direction of the given environmental variables, while the red arrows indicate the direction of species traits.

In the ordinations of species composition, all environmental predictors except for area exerted significant effects (Table 2, Figure 3). Interpreting the ordinations by the moths' traits revealed consistent patterns. The analysis constrained by altitude showed that moths inclining towards high altitudes tend to occupy wide habitat and altitude ranges, overwinter in later stages and develop on shrubs, trees or non‐plants. In contrast, moths inclining towards lowland reserves occupy narrower habitat and altitude ranges, develop on forbs and form multiple generations per year (Figure 3a). Constraining the analysis by elevational scope indicated affiliation of moths forming multiple generations, having short flight periods and developing on stems or roots towards reserves with a greater elevational scope (Figure 3b). The analysis based on geographic coordinates explained the highest percentage of explained variation. It returned a complex model directing from the southeastern and lowland positions towards high altitudes. Moths inclining towards the former are larger, develop on leaves of forbs or grasses, produce more generations per year, utilise fewer habitats, occupy narrower altitude ranges and overwinter in early developmental stages (Figure 3c).

**TABLE 2:** The results of fourth‐corner analyses relating moth species composition to reserve characteristics (Step 1) and interpreting the resulting species scores by species traits (Step 2).

	Step 1: DCCA of moths species composition				Step 2: RDA interpretation of significant DCCA axes by species traits				
	EV1	EV2	Var %	*F* ^*P*^_all_	EV1	EV2	Var %	*F* ^*P*^_all_	Interpretation
Areaǀsurveying effort	0.01	—	0.00	0.7^NS^	—	—	—	—	—
Altitudeǀsurveying effort	0.04	—	0.69	3.0***	0.05	—	4.2	9.3***	1st axis: host plant form, habitat range, overwintering state → *altitude* ← voltinism
Elevational scopeǀsurveying effort	0.02	—	0.24	1.7**	0.05	—	4.8	16.9***	1st axis: voltinism, stems/roots → *elevational scope* ← flight period length
Geography^a^ǀsurveying effort	0.07	0.02	2.58	1.9***	0.02	0.01	3.1	4.7***	1st axis: overwintering stage, habitat range, host plant form, flowers/seeds, voltinism, stem/roots → *gradient from North, West and mountains towards East and lowlands* ← wingspan, leaves
Phytogeographic regionǀsurveying effort	0.04	—	0.58	2.7***	0.050	—	4.6	12.2***	1st axis: wingspan ← *Hercynian* → altitude range, habitat range, host plant form
Phytogeographic divisionǀsurveying effort	0.06	—	1.34	3.0***	0.03	0.01	2.8	4.8***	1st axis: wingspan, voltinism ← *Oreophyticum* → host plant form, overwintering stage, habitat range, stems/roots 2nd axis: altitude range, stem/roots ← *Thermophyticum*
Biotopeǀsurveying effort	0.06	0.02	1.78	2.1***	0.01	0.01	1.8	6.7***	1st axis: wingspan← *mountain and peat bogs* → host plant form, stems/roots 2nd axis: stems/roots ← *forest* → host plant form, wingspan

*Note:* The arrows indicate which traits correlate with the axis (→) and which, on the contrary, are oriented against the axis (←). Asterisks indicate statistically significant effects (**p < 0.01, ***p < 0.001).

^a^ Forward‐selected from polynomials and multiplicative interactions: ~latitude × latitude + latitude × longitude + latitude × altitude + latitude^2 + longitude × altitude + longitude^2 + altitude^2.

Ordination constrained by phytogeographic regions characterised moths inclining towards the Hercynian region as occupying wider habitat and altitude ranges and developing on shrubs, trees and non‐plants, whereas Carpatho‐Pannonian moths display larger wingspan and development on forbs and grasses (Figure 3d). The ordination constrained by phytogeographic division distributed the moths along two gradients, one towards Thermophyticum (Axis 1) and the other towards Oreophyticum (Axis 2). The two gradients were correlated with identical traits: moths associated with both gradients are larger, form fewer generations per year, occupy narrower altitude ranges, utilise fewer habitats, overwinter in earlier stages and develop on forbs or grasses (Figure 3e).

Analysis constrained by biotopes differentiated steppes and mesic grasslands from mountain forests and peat bogs (Axis 1), and wetlands and peat bogs from mountain forests (Axis 2). Larger moths developing on forbs or grasses and inhabiting narrower altitude ranges and fewer habitats inclined towards steppes and mesic grasslands. Species developing on stems or roots of forbs and grasses inclined towards mountain forests and bogs (Figure 3f).

The elevational scope of a reserve was the only predictor increasing (marginally significantly) the number of Red‐listed species (Table 3).

**TABLE 3:** The results of fourth‐corner analyses Red‐listed moths species composition to reserve characteristics (Step 1) and interpreting the resulting species scores by species traits (Step 2).

	Step 1: DCCA of moths species composition				Step 2: RDA interpretation of significant DCCA axes by species traits				
	EV1	EV2	Var %	*F* ^*P*^_all_	EV1	EV2	Var %	*F* ^*P*^_all_	Interpretation
Presences set									
Area ǀ surveying effort	0.01	—	0	0.7^NS^	—	—	—	—	—
Altitude ǀ surveying effort	0.04	—	0.79	3.3***	0.01	—	0.08	1.1^NS^	—
Elevational scope ǀ surveying effort	0.02	—	0.24	1.7**	0.02	—	1.32	3.3 (*)	1st axis: *elevational scope* → Red list
Geography^a^ ǀ surveying effort	0.07	0.02	2.58	1.9***	0.01	—	0.06	1.1^NS^	—
Phytogeographic region ǀ surveying effort	0.04	—	0.58	2.7***	> 0.01	—	0.00	0.40^NS^	—
Phytogeographic division ǀ surveying effort	0.06	—	1.34	3.0***	> 0.01	—	0.00	0.50^NS^	—
Biotope ǀ surveying effort	0.06	0.02	1.78	2.1***	0.01	—	0.27	0.21^NS^	—

*Note:* The arrow indicates which traits correlate with the axis (→). Asterisks indicate statistically significant effects (**p < 0.01, ***p < 0.001, (*) marginally significant).

^a^ Forward‐selected from polynomials and multiplicative interactions: ~latitude × latitude + latitude × longitude + latitude × altitude + latitude^2 + longitude × altitude + longitude^2 + altitude^2.

## 4 Discussion

Our results demonstrate that moths' inventories, despite returning mere species lists and despite being based on varying field procedures, reveal robust macroecological and traits‐related patterns. Specifically, the results of 292 inventories analysed here provide a comprehensive picture of the moths' distribution patterns and their links with moths' species traits, on the scale of a medium‐sized European country.

Species richness recorded per reserve was strongly and expectably influenced by surveying effort, expressed here as the number of visits per reserve. It should be emphasised that the number of visits, as used by us, represents only a crude indication of effort. The species lists obtained from contracted reserve inventories are crucially influenced by the light sources used (Balamurugan and Kandasamy 2021), the Moon phase and the weather during the surveys (Yela and Holyoak 1997), the number of light traps and their placement across the reserve (spatial replication) and the distribution of visits in time. Practically all temperate moths display distinct adult phenological patterns (Potocký et al. 2018). Consequently, more complete coverage of seasonal aspects increases the number of species intercepted (Zapletal et al. 2026). Nevertheless, our data support the assumptions of, for example, Summerville and Crist (2002), Duran et al. (2022) or Vrba et al. (2024) that even a limited number of visits may reveal valuable information about moth communities' structure.

When accounting simultaneously for surveying effort, reserve characteristics, geographic position and phytogeographic classification, macro‐moth species richness was explained by surveying effort, elevational scope and (crudely defined) biotope, whereas altitude showed a marginally negative relationship. Elevational scope was positively related to surveying effort (Table S1), but exerted a strong positive effect on species richness even in models simultaneously accounting for surveying effort. Likely, authors of the inventories expected more diverse moths' communities in topographically heterogeneous reserves and adapted to this expectation accordingly. In contrast, reserve area correlated with effort negatively, suggesting that authors of the inventories did not increase effort in large but homogeneous reserves.

The role of elevational scope suggests that variation in macro‐moth species richness across the reserves is more closely linked to biotopes and environmental heterogeneity than to area per se. Although species–area relationships are among the most frequently reported patterns in biodiversity studies (Connor and McCoy 1979; Fattorini 2020; Pyšek et al. 2002), area may be a poor proxy for ecological opportunity in heterogeneous reserves. In such systems, environmental gradients and habitat diversity, imperfectly captured by the elevational scope proxy (cf. Álvarez et al. 2024; Bartonova et al. 2016) may be more directly relevant for species richness than area alone (e.g., Báldi 2008; Das et al. 2023; Triantis et al. 2003). It may in fact represent the underlying mechanisms of the species‐area relationship, because a larger area will contain more habitats, plus a higher diversity of microhabitat conditions (Zhang et al. 2025). In our context, reserves with wide elevational scope were typically river canyons stretching from riparian vegetation and cold forests in inversion positions up to thermophilous woodlands or grasslands at upper rims (e.g., Šumpich 2011), or mountain slopes covering multiple elevational belts (Kuras et al. 2009; Spitzer and Beneš 2023). Not surprisingly, such reserves exhibit high diversity of habitats and species.

Regarding biotopes, inventories of steppes and mesic grasslands returned more species than inventories of other biotopes. This is in line with high species richness of these habitats in Central Europe (e.g., Chytrý et al. 2015; Feurdean et al. 2018; Roleček et al. 2025; Slancarova et al. 2014), the positive link between plants' and herbivores' diversity (Futuyma and Agrawal 2009), and the fact that many such reserves do not contain a single biotope, but are rather grasslands embedded amidst woody vegetation (e.g., Walter et al. 2025). The open habitats in nemoral Europe arguably derived from megafauna‐maintained interglacial, and early post‐glacial, parklands (Pearce et al. 2023; Czyżewski et al. 2026). Their depend on active management measures at present (Šumpich and Konvička 2012; Percel et al. 2025) and their high species richness underlines the importance of the measures.

In the ordination analyses of species composition, the explained variation was generally low, ≤ 3%, but the patterns were still highly significant; such situations are common in ordinations of samples consisting of high numbers of species (O'Connell‐Booth et al. 2025; Vrba et al. 2024; Zapletal et al. 2026). Geographic position was the strongest species composition predictor, followed by biotope type, and phytogeographic division. A strong role of longitudinal and latitudinal gradients, which covary with climate, topography and bedrock composition, has previously been documented for birds and butterflies even within the relatively small spatial extent of the Czech Republic (Storch et al. 2003). Differences between the warmer and topographically more rugged Carpatho‐Pannonian region are well known for plants (Kaplan 2017) and consistent with recent macroecological syntheses (Šímová et al. 2023).

Regarding species traits, reserves in higher altitudes hosted more likely moths developing on shrubs, trees or non‐plants, occupying wider altitude and habitat ranges, overwintering as pupae or adults, and forming single annual generation. Most of the high‐altitude reserves were mountain forests, characterised by abundant canopy but species‐poor herb layers. This combination of traits echoes the host plant apparency theory (Ali and Agrawal 2012; Feeny 1976), which states that ‘unapparent’ forbs tend to be qualitatively protected, forcing their herbivores to utilise narrow taxonomic scope of host plants, but allowing them rapid development. In contrast, feeding on bulky and persistent quantitatively protected grasses, woody plants or non‐plants allows lower dietary specialisation but requires longer development (Seifert et al. 2023). Due to wider taxonomic trophic ranges, herbivores developing on apparent hosts may display wider habitat ranges. In contrast, most of grassland reserves are situated in lower altitudes, and grassland moths are often associated with unapparent forbs, which results in narrower habitats and altitude ranges. Finally, overwintering in later stages (here, e.g., *Cleora cinctaria*, *Xestia speciosa* as pupae; *Dasypolia templi*, *Xylena vetusta* as adults) allows relatively earlier seasonal onset of adult flight, which may be crucial in mountains (cf. Börschig et al. 2013).

Complementarily, the wider habitat ranges of moths recorded in higher altitudes might be due to the fact that mountain forests, besides high altitude specialists, also accommodate relatively generalist forest moths with wide climatic tolerance (e.g., *Biston betularia* , *Colocasia coryli*, *Spinx pinastri*). Although habitat specialisation of insects decreases with elevation (Classen et al. 2015; Richter et al. 2024; Zou et al. 2016), inventories of mountain reserves returned mixtures of mountain forest specialists and widely distributed forest generalists.

Similarly to species richness, the elevational scope proxy for habitat heterogeneity was a strong species composition predictor. Vertically heterogeneous reserves hosted moths that form multiple annual generations, develop on plant stems and roots, yet have relatively short flight periods. The combination of multiple generations and short total flight period appears contra‐intuitive, as in generalist insects, these two traits are often positively correlated (Bartonova et al. 2014; Potocký et al. 2018). Possibly, the wide vertical range allows multivoltine specialists to compensate for seasonally changing microclimates or host plant quality by short‐range vertical dispersal. This conjecture is supported by the positive association between elevational scope and Red‐listed moths, indicating that morphologically heterogenous sites function as refugia, buffering against anthropogenic pressures or climatic fluctuations (Keppel et al. 2012; Maes et al. 2024; Sanders et al. 2007; Stein et al. 2014). Note, however, that surveying effort was higher for reserves with wider elevational scope, which might increase the chance of intercepting rare taxa. Still, some Red‐listed moths may depend on geomorphological diversity per se—examples include *Entephria infidaria*, associated with rocky habitats in mountains or deep valleys (Čelechovský 2004); or *Odontognophos dumetata*, inhabiting xerophilous screes (Šumpich et al. 2013). Topographic heterogeneity thus, besides contributing to overall species richness, influences the distribution of life‐history strategies across the landscape (Hoiss et al. 2012; Rahbek and Graves 2001; Toko et al. 2023).

The ordination based on geography coordinates returned rather complex model, which reflected the complex topography of the Czech Republic, with warm lowlands situated in Southeast and Northwest, and cold mountains in the North, South and West (Kaplan 2017; cf. Figure 1). As in the model with altitude only, high altitudes (and cold regions) were associated with moths feeding on shrubs, trees and non‐plants and inhabiting wide altitude and habitat ranges, forming low numbers of generations and overwintering as pupae or adults. Low altitudes and eastern regions, on the other hand, hosted larger moths overwintering in early stages and developing on leaves. Indeed, the largest European moth, *Saturnia pyri*, occurs mainly in the Pannonian region in Central Europe, and many other large moths (e.g., *Euplagia quadripunctaria* or *Hyles euphorbiae* ) prefer warm regions. Overwintering in early stages facilitates earlier onset of larval feeding, and hence reaching large body size (cf. Seifert et al. 2023, 2025).

Because phytogeographic regions and divisions are essentially alternative expressions of geographic position (Skalický 1988), the patterns of moths' species composition reflected the above patterns. The traits associated with phytogeographic regions were identical to those associated with altitude, while those distinguishing Hercynian and Carpatho‐Pannonian division pointed to wider habitats and altitude ranges associated with the former, and narrower habitats and altitude ranges, but also larger wingspans, associated with the latter.

The ordinations with biotope types reflect all the themes discussed above. The main gradient distinguished xeric and mesic grasslands from mountain forests; the secondary gradient distinguished wetlands from forests. The traits associated with grasslands were large wingspan and development on forbs and grasses; those associated with forests were developing on trees, shrubs or non‐plants, and/or consuming stems or roots. Migratory moths were also associated with xeric grasslands, likely due to the steady influx of southern migrants to warm regions during the last decades (cf. Sparks et al. 2007). This, however, does not exclude flyovers of southern migrants across mountain passes (Hawkes et al. 2023).

It should be noted that some ecological traits may be plastic within species, varying geographically (e.g., host plant use) or with environmental conditions (e.g., smaller body size towards species range edges, or reduced number of generations in mountains) (Meister et al. 2015; Fält‐Nardmann et al. 2017). Potocký et al. (2018), the source of trait states used here, were explicitly aware of this, and tabularised trait states for lowlands of the Czech Republic. It should hence suffice for deciphering main traits responses to environment, although more detailed traits coding might bring further insights.

Inconsistent reporting of abundances in the inventories (real numbers of individuals, deliberate abundance scales or no abundance information at all) restricted us to working with species presences only. Our approach thus did not grasp abundance variation, for example, for widely distributed species that still have optima in certain biotope types. Encouragingly, this limitation did not mask such traits ~ environment relationships as developing on woody plants in forests. Many moths feeding on tree and shrub foliage reach high abundances in forests, but can be intercepted elsewhere as well (e.g., Schwerdtfeger 1952; Van Dongen et al. 1998). Their association with forests in our results thus implies that the surveys still detected such moths more likely in habitats where they occur abundantly.

Overall, the key gradient structuring moths' assemblages in the Czech Republic reserves are geographic position (which can be approximated by established division of the country into Hercynian and Carpatho‐Pannonian regions), altitude and topographic heterogeneity. Increasing altitude straightforwardly increased the chance of developing on apparent host plants, low generation number, large wingspan, short flight period and overwintering in late stages (Bartonova et al. 2014; Čížek 2013; Foerster et al. 2024; Seifert et al. 2025). The positive role of topographic heterogeneity for Red‐listed suggested the presence of diverse habitat conditions. The traits associated with those gradients were highlighted in other studies as well. Thus, Mattila et al. (2006, 2008, 2009) detected higher extinction risks in moths developing on forbs, Valtonen et al. (2017) observed more restricted distribution in trophic specialists, and Merckx et al. (2018) reported that larger moths respond more positively to urbanisation.

Despite their heterogeneous character, data obtained during macro‐moths inventories of reserves revealed meaningful ecological patterns, demonstrating the utility of such data for ecological analyses. The dataset used here, and similar data generated by conservation practice, warrants more detailed examination focusing on associations of individual species with more narrowly defined habitat types, and identification of habitats of particular importance for species of conservation concern. Such analyses would greatly benefit if the inventories, typically carried out by amateur lepidopterists, were conducted in a more standardised manner.

## Author Contributions

**Zuzana Kubincová:** conceptualization (equal), data curation (equal), formal analysis (equal), funding acquisition (equal), investigation (equal), methodology (equal), visualization (equal), writing – review and editing (equal). **Zdeněk Faltýnek Fric:** conceptualization (supporting), formal analysis (supporting), investigation (supporting), methodology (supporting), writing – original draft (supporting), writing – review and editing (supporting). **Radek Hejda:** conceptualization (equal), data curation (equal), formal analysis (supporting), investigation (supporting), methodology (supporting), writing – original draft (supporting), writing – review and editing (supporting). **Martin Konvička:** conceptualization (equal), data curation (supporting), formal analysis (equal), investigation (equal), methodology (equal), supervision (lead), visualization (supporting), writing – original draft (lead), writing – review and editing (lead). **Lukáš Spitzer:** conceptualization (supporting), formal analysis (supporting), investigation (supporting), methodology (supporting), writing – original draft (supporting), writing – review and editing (supporting). **Alena Sucháčková:** conceptualization (supporting), formal analysis (supporting), investigation (supporting), methodology (supporting), writing – original draft (supporting), writing – review and editing (supporting). **Pavel Vrba:** conceptualization (supporting), formal analysis (supporting), investigation (supporting), methodology (supporting), writing – original draft (supporting), writing – review and editing (supporting). **Jan Walter:** conceptualization (equal), data curation (lead), formal analysis (lead), investigation (equal), methodology (equal), supervision (lead), visualization (lead), writing – original draft (lead), writing – review and editing (lead).

## Funding

This study was supported by BAMAPE 2025 (BAMAPE 2025‐06) at the Faculty of Education, University of West Bohemia, and by the Technology Agency of the Czech Republic (SS07020400).

## Conflicts of Interest

The authors declare no conflicts of interest.

## Supporting information


**Table S1:** Ordinal logistic regression of surveying effort (dependent variable, coded as ordinal factor) against reserve characteristics.
**Figure S1:** Standardised effects of all reserve characteristics on macro‐moth species richness derived from a multiple linear regression model. Points represent standardised regression coefficients, and horizontal bars indicate 95% confidence intervals. The dashed vertical line denotes zero effect. Asterisks indicate statistically significant effects (*p < 0.05, **p < 0.01, ***p < 0.001). All continuous predictors were standardised to zero mean and unit variance prior to analysis.

**Data S1:** Data on reserve characteristics, species occurrence, and species traits.

## Data Availability

All the required data are uploaded as Supporting Information.
