# Supplementary material for: From Inventories to Insights: Environmental Gradients Structuring Macro‐Moths Assemblages Recorded in Nature Reserves
Source: Ecol Evol. 2026 Jun 8;16(6):e73788. doi: 10.1002/ece3.73788 (PMC13244079; doi:10.1002/ece3.73788)

**Supplementary materials**

**Table S1.** Ordinal logistic regression of surveying effort (dependent variable, coded as ordinal factor) against reserve characteristics

R-code: model <- clm(visits ~ altitude +longitude +elevational scope +latitude +area+ phytogeographic regions + phytogeographic divisions +biotope, data = (data), link = "logit")

number of observations: 292, logLikelighood: -235.0, AIC: 500.0, number of iterations: 5(0), max grad.: 7.62e^-8^, conditional H: 8.5e^+01^

Estimates of model coefficients and significance tests.

|  | Estimate | SE | z-value | P |
| --- | --- | --- | --- | --- |
| area | -0.36 | 0.178 | -2.05 | * |
| altitude | -0.05 | 0.214 | -0.24 | 0.81 |
| elevational scope | 0.75 | 0.174 | 4.31 | *** |
| longitude | -0.04 | 0.212 | -0.19 | 0.85 |
| latitude | -0.27 | 0.159 | -1.67 | 0.09 |
| phytogeographic regions: Hercynia | -0.79 | 0.480 | -1.65 | 0.10 |
| phytogeographic divisions: Oreophyticum | 0.17 | 0.497 | 0.34 | 0.74 |
| phytogeographic divisions: Thermophyticum | 0.46 | 0.380 | 1.20 | 0.23 |
| biotope: mesic grasslands | 0.22 | 0.589 | -0.37 | 0.71 |
| biotope: mountain forests | -0.31 | 0.597 | -0.52 | 0.60 |
| biotope: peat bogs | 0.29 | 0.536 | 0.54 | 0.59 |
| biotope: steppes | -0.25 | 0.379 | -0.66 | 0.51 |
| biotope: wetlands | 0.09 | 0.391 | 0.22 | 0.83 |

**Figure S1.** Standardised effects of all reserve characteristics on macro-moth species richness derived from a multiple linear regression model. Points represent standardised regression coefficients, and horizontal bars indicate 95% confidence intervals. The dashed vertical line denotes zero effect. Asterisks indicate statistically significant effects (* p < 0.05, ** p < 0.01, *** p < 0.001). All continuous predictors were standardised to zero mean and unit variance prior to analysis.
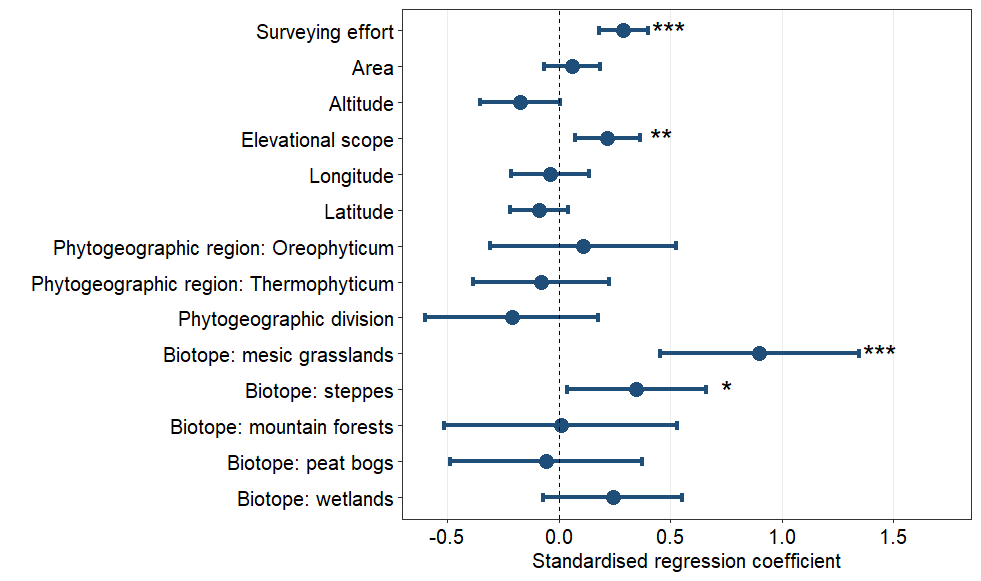

Supplement: Supplementary file 1 — Table S1: Ordinal logistic regression of surveying effort (dependent variable, coded as ordinal factor) against reserve characteristics. Figure S1: Standardised effects of all reserve characteristics on macro‐moth species richness derived from a multiple linear regression model. Points represent standardised regression coefficients, and horizontal bars indicate 95% confidence intervals. The dashed vertical line denotes zero effect. Asterisks indicate statistically significant effects (*p < 0.05, **p < 0.01, ***p < 0.001). All continuous predictors were standardised to zero mean and unit variance prior to analysis. [file ECE3-16-e73788-s002.docx]
